# Patient outcomes following surgical management of multinodular goiter: Does multinodularity increase the risk of thyroid malignancy?

**DOI:** 10.1097/MD.0000000000004194

**Published:** 2016-07-18

**Authors:** Yann-Sheng Lin, Hsin-Yi Wu, Ming-Chin Yu, Chih-Chieh Hsu, Tzu-Chieh Chao

**Affiliations:** a Department of Surgery, Chang Gung Memorial Hospital, Linkou; b Chang Gung University, Kwei-Shan Tao-Yuan, Taiwan.

**Keywords:** cancer multifocality, incidental carcinoma, multinodular goiter, multinodularity, thyroidectomy, total

## Abstract

**Background:**

the difference in the risk of thyroid malignancy for patients with multinodular goiter (MNG) and solitary nodular goiter (SNG) remains controversial. Although total thyroidectomy (TT) is the current preferred surgical option for MNG, permanent hypothyroidism in these patients may be a concern. Therefore, we discuss whether nontotal thyroidectomy is a reasonable alternative surgical option.

**Methods:**

A retrospective cohort study was performed for 1598 consecutive patients who underwent thyroid surgery for nodular goiter between January 2007 and December 2012. Numerous clinical parameters were collected and analyzed.

**Results:**

We reviewed 795 patients with MNG and 803 patients with SNG. The prevalence of malignancy on final pathology was significantly higher in the patients with MNG than in the patients with SNG (15.6% vs 10.1%, *P* = 0.001). However, a multivariate analysis revealed that this difference was insignificant (*P* = 0.50). Papillary carcinoma was the predominant type in both groups, but papillary microcarcinoma was more frequently found (41.1%) in the patients with MNG. The only multifocal cancers were of the papillary carcinoma histologic type, and the incidence of multifocal papillary carcinoma was significantly higher in the patients with MNG (23.4% vs 7.4%, *P* = 0.005). Reoperation was not required for the patients who underwent TT for goiter recurrence or incidental carcinoma. The overall rate of recurrence following nontotal thyroidectomy was 12.2%. Among the patients who underwent reoperation for goiter recurrence, 2 (20.0%) were complicated with permanent hypoparathyroidism. Among the patients who underwent a nontotal bilateral thyroidectomy, an average of 56.5% had permanent hypothyroidism.

**Conclusions:**

Multinodularity does not increase the risk of thyroid malignancy. However, patients with MNG who develop papillary carcinoma are at an increased risk of cancer multifocality. If a patient can tolerate lifelong thyroid hormone replacement, TT is the preferred surgical option because it helps avoid reoperation and the associated complications. Nontotal bilateral thyroidectomy does not ensure the preservation of thyroid hormone function.

## 1 Introduction

Multinodular goiter (MNG) is defined as an enlarged thyroid gland with multiple nodules and occurs in up to 4% of the population in iodine-sufficient countries, and its frequency increases with age.^[1]^ The incidence of MNG is in the range of 10% to 20% when ultrasound is used as a screening tool and can be as high as 70% when using high-resolution ultrasound.^[2]^ Surgeons frequently encounter this disease in their daily practice. Patients with MNG may undergo surgery to address goiter enlargement, which can cause compressive symptoms or cosmetic concerns; thyrotoxicosis; indeterminate cytology and suspicious or diagnosed malignancy. Currently, total thyroidectomy (TT) is the preferred option for bilateral MNG because a complete resection avoids further surgeries for incidental thyroid cancer (as diagnosed by final pathology results) or disease recurrence.^[3–5]^ However, permanent hypothyroidism is a concern for patients because it necessitates lifelong hormone replacement therapy (HRT). In this study, we discuss whether nontotal thyroidectomy is a reasonable alternative surgical management option.

The risk of malignancy in patients with MNG remains controversial. Previous research using ultrasound-guided fine-needle aspiration biopsy (FNAB) as a diagnostic tool reported that the prevalence of malignancy in patients with MNG was identical to that in patients with SNG.^[6–8]^ However, recent studies have reported that the risk of carcinoma in patients with MNG has been underestimated, and a prevalence in the range of 17% to 35% has been reported in surgical series.^[9–11]^ A meta-analysis by Brito et al^[12]^ revealed that thyroid cancer may be less frequent in MNG than in SNG, particularly in iodine-deficient populations. Therefore, we will also discuss whether there are variations in the risk of malignancy with respect to final pathology and histopathologic features of patients with MNG versus SNG.

## 2 Materials and methods

**Figure 1:**
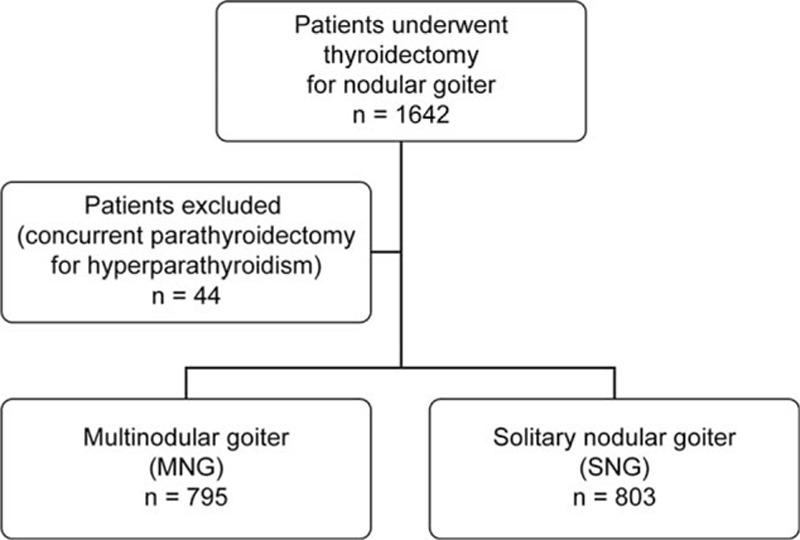
Flowchart illustrating the study design.

This retrospective cohort study was approved by the Institutional Review Board (IRB) of the Chang Gung Memorial Hospital, Linkou, Taiwan (IRB Reference Number: 104-6213B). Between January 2007 and December 2012, 1642 patients with a diagnosis of nodular goiter by sonography who underwent thyroidectomy were identified. Indications for surgery included compressive symptoms, cosmetic concerns, thyrotoxicosis, indeterminate cytology (including atypia, follicular neoplasm, and Hurthle cell neoplasm) and suspicious or proved malignancy by FNAB. Forty-four patients were excluded because of concurrent parathyroidectomy for hyperparathyroidism. Finally, 1598 patients were enrolled in this study. MNG was defined as 2 or more nodules observed on preoperative ultrasound. Based on this definition, 795 patients were diagnosed with MNG, and 803 patients were diagnosed with SNG (Fig. 1). Their medical records were reviewed, and data were collected and analyzed. The last follow-up visit was in December 2015.

An ultrasound of the neck was routinely conducted on each patient. We followed the American Thyroid Association guidelines^[13]^ for performing an FNAB on the dominant nodule, which was defined as the largest and most prominent nodule by sonography or the nodule with suspicious sonographic features (i.e., hypoechoic lesion, ill-defined margins, microcalcification in the nodule, or increased vascularity). Incidental carcinoma was defined as malignancy (as confirmed by the final pathological examination) that presented with a lack of suspicious features on preoperative ultrasound or FNAB. During the study period, completion thyroidectomy was suggested for patients with a tumor >1 cm in diameter, extrathyroidal extension, node positivity, or multifocality evident in the resected specimen. Cancer multifocality was defined as 2 or more tumor foci located in the same lobe. Papillary carcinoma was further divided into microcarcinoma, defined as a tumor size <1 cm in diameter, and macrocarcinoma, defined as a tumor size >1 cm in diameter.

Serum calcium and parathyroid hormone (PTH) concentrations were routinely measured on postoperative day 1 for the patients who underwent bilateral thyroidectomy. These tests were also conducted on the patients who underwent unilateral thyroidectomy and had hypocalcemia symptoms. Symptomatic hypocalcemia was defined as a serum calcium concentration <8.0 mg/dL with subjective symptoms, including paresthesia of the distal limbs, tetany, or carpopedal spasm. Permanent hypoparathyroidism was defined as having a postoperative PTH concentration <14 pg/mL over 6 months.

Goiter recurrence was defined as a relapse occurring at the surgical site as detected by ultrasound during the postoperative follow-up. Nodules found in the contralateral lobe that were not resected in the initial surgery were defined as retained lesions. Postoperatively, thyroxine was used for HRT in patients who underwent TT or when hypothyroidism was identified biochemically. Thyroxine was not used for thyroid-stimulating hormone (TSH) suppression in patients with remnant thyroid and a benign pathological diagnosis, although TSH suppression was indicated for patients with a pathological diagnosis of malignancy.

The Mann–Whitney *U* test or Kruskal–Wallis *H* test was performed for continuous variables. Group comparisons of nominal data were achieved using Pearson's chi-squared (χ^2^) test or Fisher's exact test if an observed value was <5. Multiple logistic regression analysis was used for the multivariate analysis. SPSS v. 22.0 (IBM Corp., Armonk, NY) was used for all analyses. All statistical tests were 2-sided, and a *P-*value of < 0.05 was considered statistically significant.

## 3 Results

### 3.1 General patient data

The demographic data, operative methods, and pathological diagnoses of the patients with MNG and SNG are shown in Table 1. The mean age of the patients with MNG at the time of surgery was 52.4 ± 12.4 years, which was significantly older than that of the patients with SNG. There was no significant difference regarding surgical indication between the groups. The operative method was significantly different between the patients with MNG and those with SNG. Most of the patients (79.0%) with MNG underwent bilateral thyroidectomy. However, the majority of the patients (96.0%) with SNG underwent unilateral thyroidectomy (UT). Among the patients with MNG, 167 cases (21.0%) having apparent unilateral goiter underwent UT, including 151 patients who underwent lobectomy and 16 who underwent unilateral subtotal thyroidectomy because all nodules were in the same lobe or the nodules in the contralateral lobe were <1 cm in diameter and lacked clinical significance.

**Table 1:**
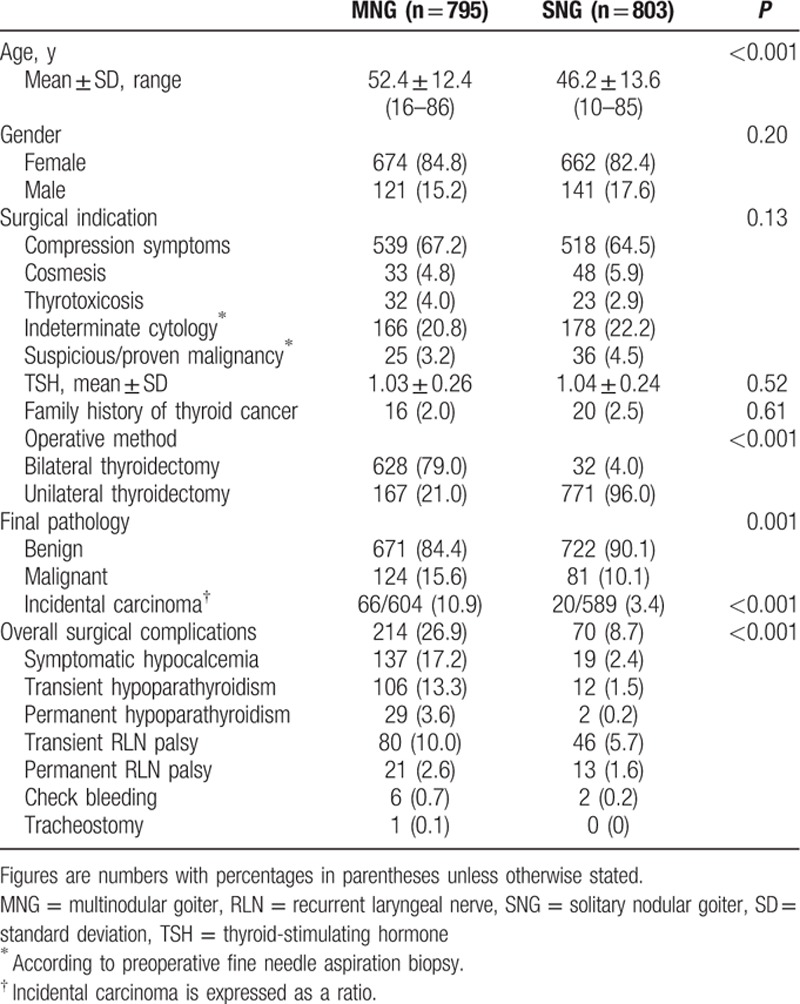
Univariate analysis of characteristics between the patients with MNG and those with SNG.

The overall complication rate was significantly higher for the patients who underwent thyroidectomy for MNG. The majority of complications following thyroidectomy for MNG were transient, including symptomatic hypocalcemia (17.2%), followed by transient hypoparathyroidism (13.3%), and transient recurrent laryngeal nerve (RLN) palsy (10.0%). One patient who underwent TT for MNG experienced bilateral RLN palsy, and an emergency tracheostomy was performed for acute respiratory distress. There were no surgical mortalities in our series.

The malignancy rate on final pathology was significantly higher in the patients with MNG than in the patients with SNG (15.6% vs 10.1%, *P* = 0.001). Incidental carcinoma also occurred more frequently in the MNG patients (10.9% vs 3.4%, *P* < 0.001), and 81.8% (54/66) of the incidental carcinoma was papillary microcarcinoma. Furthermore, we performed a multivariate analysis of the confounding factors, including patient age and operative method, to identify if the risk of malignancy was different between the patients with MNG and those with SNG. We found that patient age and operative method were significantly different between the 2 groups, both with *P* values < 0.001. However, the difference in malignancy rate on final pathology was insignificant (*P* = 0.50).

### 3.2 Preoperative FNAB

Among the patients with MNG, ultrasound results revealed 2 nodules in 205 patients (25.8%), 3 nodules in 301 patients (37.8%), 4 nodules in 197 patients (24.8%), and ≥ 5 nodules in 92 patients (11.6%). A preoperative ultrasound-guided FNAB was performed for 700 patients with MNG. FNAB was not repeated in 95 patients because of a benign cytology report from the referring institution. We excluded patients with indeterminate FNAB results (n = 166). Subsequently, the numbers of true-positive and false-negative patients were 11 and 66, respectively. The sensitivity, specificity, positive predictive value, and negative predictive value of the FNAB were calculated as 14.3%, 96.9%, 44.0%, and 87.0%, respectively. In the meantime, FNAB was performed preoperatively for 614 patients with SNG in our hospital; the sensitivity, specificity, positive predictive value and negative predictive value were 94.1%, 98.9%, 88.8%, and 99.5%, respectively.

### 3.3 Comparison between different operative methods for MNG

The patients were divided into 4 groups according to the operative method (Table 2). The patients who underwent TT were significantly older, and the operative time and intraoperative blood loss were significantly greater in the patients who underwent bilateral subtotal thyroidectomy (BST). The patients who underwent bilateral resection had a relatively longer hospital stay. TT had a significantly higher transient complication rate, except for transient RLN palsy. However, statistically significant differences were not observed for permanent complications. Completion surgery was not required in the TT group. The rate of postsurgical permanent hypothyroidism was 63.3% following the Dunhill operation (DO), 41.4% following BST, and 2.4% following UT.

**Table 2:**
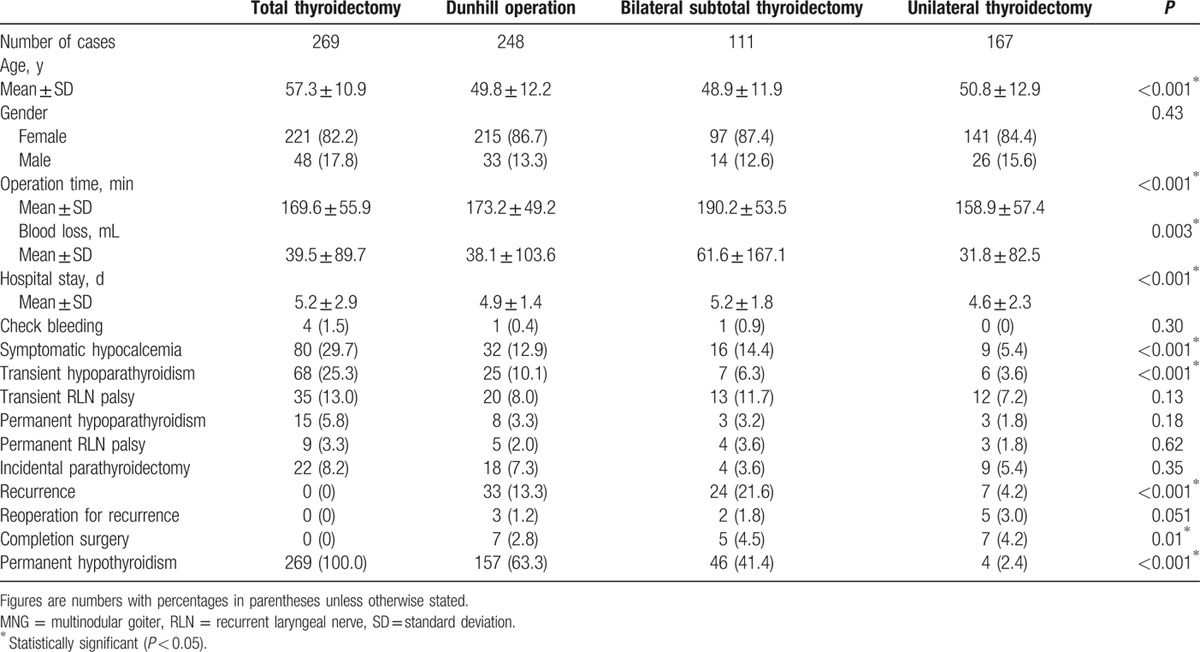
Comparison between operative methods for MNG.

### 3.4 Patient follow-up

After a mean follow-up time of 53.8 ± 34.0 months (range: 3–108 months), goiter recurrence was observed in 64 patients who underwent nontotal thyroidectomy (Table 2). The overall rate of recurrence following nontotal thyroidectomy was 12.2% (64/526). Among the patients with goiter recurrence, 10 cases (15.6%) required a secondary operation because an ultrasound revealed that the goiter had grown in size and was associated with compressive symptoms. The pathology results indicated that all specimens were benign. Two of them (20.0%) were complicated with permanent hypoparathyroidism. Among the 167 patients who underwent UT, recurrence was not observed in those who underwent complete lobectomy. However, recurrence was identified in 7 patients who underwent unilateral subtotal thyroidectomy. In the UT group, 42 patients (25.1%) with benign pathology retained nodules in the contralateral lobe. During follow-up, growing nodules were detected by ultrasound in 10 patients (23.8%); only 1 required additional surgery to remove the contralateral lobe because of compressive symptoms.

### 3.5 Histopathologic features

The histologic types of the malignancies identified in the patients with MNG or SNG are shown in Table 3. Papillary carcinoma was the predominant type in both groups, although the ratio of papillary to follicular carcinoma (including Hurthle cell carcinoma) increased from 3.3 (63/19) in the patients with SNG to 8.5 (110/13) in the patients with MNG (*P* = .02). Papillary microcarcinoma was more frequently found in the patients with MNG, and the majority (54/56) of cases were found incidentally in the submitted specimens. The only multifocal cancers were of the papillary carcinoma histologic type, and the incidence of multifocal papillary carcinoma was significantly higher in the patients with MNG (23.4% vs 7.4%, *P* = 0.005). Among this group, cancers occurring in the bilateral lobes were found in 24 patients (19.4%), with the majority presenting bilateral papillary carcinoma bilaterally and only 2 presenting simultaneous follicular and papillary carcinomas in separate lobes. There was no significant difference in the mean tumor size between the groups (*P* = 0.07). Among the patients with MNG, the mean tumor size of the incidental tumor foci was significantly smaller than the dominant nodules as observed on ultrasound (0.67 ± 0.58 cm vs 2.49 ± 1.67 cm, *P* < 0.001). Completion surgery was required for 19 patients with MNG who initially underwent nontotal thyroidectomy. Among them, 10 patients (52.6%) underwent surgery because of cancer multifocality and 1 was complicated by permanent RLN palsy.

**Table 3:**
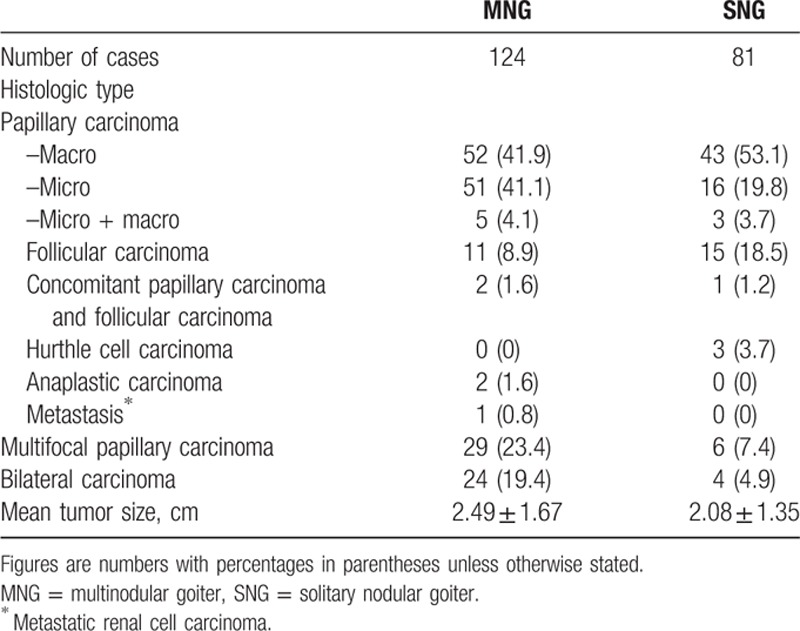
Histopathologic features of malignancies in the patients with MNG and those with SNG.

## 4 Discussion

Current series have demonstrated that total thyroidectomy is effective for MNG treatment.^[4,5,10,14]^ One of the most important supporting factors for this procedure is the virtual absence of recurrence after TT (rate: 0–0.3%).^[15]^ Although the transient complication rate of TT was higher than other operative methods for MNG, there was no difference in the permanent complication rate. The recurrence rate after nontotal thyroidectomy among published series has varied from 5% to 40% because different clinical definitions, patient populations, primary surgical procedures, and follow-up durations were applied. In this study, when additional thyroid remnants were retained during initial surgery, the incidence of recurrence increased, with a rate of 13.3% following DO and up to 21.6% following BST. Among patients with recurrence, reported rates of reoperation are also highly variable and range from 4% to 89%.^[16,17]^ The incidence of complications after reoperation was considerably higher than that after the primary operation because of tissue scarring and fibrous adhesions.^[18,19]^ Among our patients who underwent reoperation for goiter recurrence, permanent hypoparathyroidism was observed in 2 patients (20.0%), which was a significantly higher incidence than that after the primary surgery. Although not all patients with recurrence require additional surgery, an increased risk of morbidity is observed with further surgery.

Permanent hypothyroidism following TT may be of concern for patients with MNG because of the cost of lifelong HRT. However, 56.5% of the patients managed by nontotal bilateral resection developed permanent hypothyroidism, which translated to a range of 41.4% to 63.3% depending on the extent of resection. Based on our observations, nontotal bilateral thyroidectomy does not ensure the preservation of thyroid hormone production.

Several authors have discussed the rationale for UT in patients with MNG having apparent unilateral nodules.^[20–22]^ Consistent with the data from Olson et al^[20]^ and Bauer et al,^[22]^ we agree that UT should be performed for unilateral benign MNG because it presents with a lower surgical morbidity than bilateral resection and a lower probability of lifelong HRT. In the present study, permanent hypothyroidism occurred in 4 patients (2.4%) following UT, and the leading cause was strongly related to the underlying thyroid condition, with 2 patients presenting Hashimoto's thyroiditis and 2 patients presenting contralateral lobe atrophy.

The recurrence rate after UT has recently been reported to range from 2% to 11%.^[20–22]^ In our series, the recurrence rate after UT was 4.2%, and all cases of recurrence occurred in patients who initially underwent unilateral subtotal thyroidectomy. Furthermore, 5 of these 7 patients (71.4%) required secondary surgery for a growing goiter associated with compressive symptoms. In the patients with retained nodules in the contralateral lobe, the majority (76.2%) of nodules remained stable in size during follow up. Only 1 patient required reoperation for a growing goiter with compression. According to these findings, if UT is considered the initial surgical option in patients with apparent unilateral MNG, complete lobectomy should be performed.

According to our data and the reports of Ríos et al^[23]^ and Al-Yaarubi et al,^[24]^ FNAB is not useful for differentiating between benign and malignant goiters in patients with MNG because of its low sensitivity rate of 14% to 17%. The possible reason for this may be related to tumor foci that are too small to enable a preoperative diagnosis. The pathological analysis in the present study revealed that the mean size of the incidental tumor foci was significantly smaller than that of the dominant nodules. Smaller tumors nesting in a large goiter or a confluence of nodules hamper the evaluation of sonographic features.^[25]^ Additionally, taking a biopsy of every nodule in MNG may not be practical, and such difficulties could explain the high rate of incidental carcinoma in MNG following thyroidectomy for benign thyroid diseases.

The propensity for multifocal papillary carcinoma, a potential risk factor for disease recurrence,^[26–28]^ was high in the patients with MNG in our series. The patients with MNG who developed papillary carcinoma had an increased risk of multifocal cancers compared with the patients with SNG who developed papillary carcinoma (odds ratio = 1.805, 95% confidence interval: 1.340–2.431, *P* < 0.001). As mentioned earlier, cancer multifocality was the main reason for completion surgery, and 1 patient experienced permanent RLN palsy. Based on these findings, TT appears a reasonable treatment option for bilateral MNG because it spares patients from having to undergo completion thyroidectomy, either for incidental carcinoma or multifocal cancers, and avoids the associated complications.

**Figure 2:**
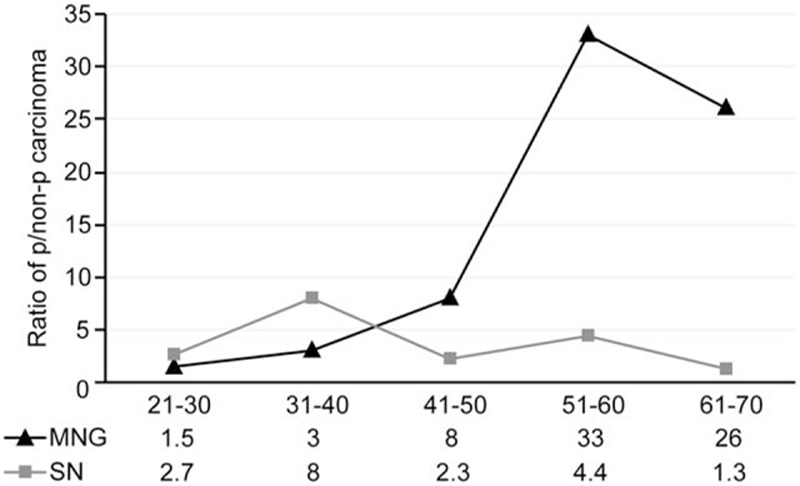
Ratio of papillary to nonpapillary carcinoma according to patient age distribution.

The prevalence of malignancy on final pathology in the patients with MNG was significantly higher than in the patients with SNG. However, a multivariate analysis revealed this difference to be statistically insignificant, which may be explained by the age of the patient population. According to the Surveillance, Epidemiology, and End Results database (1975–2012), the incidence of thyroid malignancy increases steadily with age regardless of sex, race, or histology.^[29]^ The mean age of our surgical patients with MNG was significantly greater than that of our patients with SNG. Furthermore, we analyzed the ratio of papillary to nonpapillary carcinoma corresponding to the patient age distribution, although patients <20 years and >70 years were excluded because only 1 subset of histologic malignancy was found. Our results indicated that the ratio significantly increased in the patients with MNG who were >40 years of age (Fig. 2), whereas the ratio for the patients with SNG remained steady. Apart from the patient age factor, an additional explanation for the results may be related to the difference in the extent of the surgical resection. The majority of the patients with MNG underwent thyroidectomy involving the bilateral lobes. Additional thyroid tissue was obtained for the pathological examination, which may have led to the increased identification of malignancy. Consistent with other surgical series,^[9,10,24]^ papillary microcarcinoma was the predominant histologic type on final pathology, with a percentage range from 41% to 67%. If we considered papillary microcarcinoma without clinico-pathological evidence of metastasis or extrathyroidal extension a benign disease, then there was no significant difference in the prevalence of malignancy between the patients with MNG and SNG (9.2% [73/795] vs 8.1% [65/803], *P* = 0.49).

## 5 Limitations

During our review of preoperative ultrasound examinations for MNG, we found that most ultrasound operators recorded the major large nodule or the dominant nodule in their reports without mention of every nodule in detail. Moreover, most of the biopsies were taken from nodules > 1.0 cm in size. This could lower the accuracy of FNAB when evaluating MNG. Another limitation was the length of the follow-up investigation, which may have influenced the recurrence rate assessment.

## 6 Conclusions

In patients with nodular goiter, the risk for malignancy is the same for those with MNG and SNG when the effects of patient age and the extent of surgical resection are excluded. However, patients with MNG who develop papillary malignancy have an increased risk of cancer multifocality. Surgical management of MNG should be individualized. In the case of bilaterally distributed MNG, if a patient can tolerate lifelong HRT, TT is the preferred option because it helps avoid reoperations, either for recurrent goiters or incidental carcinoma, which require additional surgery and have various associated complications. Nontotal bilateral thyroidectomy does not ensure the preservation of thyroid hormone function. Therefore, patients must be preoperatively informed about the risk of this choice. For patients with MNG with apparent unilateral nodules, complete lobectomy rather than unilateral subtotal thyroidectomy is recommended as the initial surgical option.
